# Effects of the active parts of Yangxin Tongmai Formula on myocardial injury and spatial metabolic remodeling in rats with myocardial ischemia-reperfusion injury

**DOI:** 10.3389/fcvm.2026.1857561

**Published:** 2026-07-20

**Authors:** Di Ye, Yi Wang, Ziyuan Zhao, Caiyue Lin, Wenhua Xu, Jinghui Zheng

**Affiliations:** 1 Graduate School, Guangxi University of Chinese Medicine, Nanning, China; 2 Academic Affairs Office, Guangxi University of Chinese Medicine, Nanning, China

**Keywords:** active parts of Yangxin Tongmai Formula, blood stasis syndrome, MALDI-MSI, myocardial ischemia-reperfusion injury, spatial metabolic remodeling

## Abstract

**Objective:**

To investigate the protective effects of the active parts of Yangxin Tongmai Formula (apr-YTF) against myocardial ischemia-reperfusion injury (MIRI) in rats from the perspective of spatial metabolic remodeling.

**Methods:**

A rat model of MIRI with blood stasis syndrome was established. Rats were randomized into Control, Model, Vehicle, and apr-YTF groups. apr-YTF was administered by gavage for 14 days. Cardiac function and myocardial injury were assessed by echocardiography, serum myocardial enzyme assays, triphenyltetrazolium chloride (TTC) staining, and hematoxylin and eosin (HE) staining. Matrix-assisted laser desorption/ionization mass spectrometry imaging (MALDI-MSI) was used to analyze the spatial distribution patterns and metabolic profiles of differential metabolites in myocardial tissue.

**Results:**

apr-YTF significantly improved left ventricular ejection fraction (LVEF) and left ventricular fractional shortening (LVFS), reduced serum creatine kinase (CK), creatine kinase-MB isoenzyme (CK-MB), lactate dehydrogenase (LDH), and lactate dehydrogenase 1 (LDH1) levels, decreased infarct size, and alleviated myocardial fibrosis and inflammatory infiltration. MALDI-MSI identified 1,282 metabolites, including 702 MIRI model-associated differential metabolic features, mainly including amino acids, nucleotides, and glycerophospholipids. The MIRI group exhibited extensive metabolic remodeling involving lipid, amino acid, nucleotide metabolism, and cofactor biosynthesis pathways. After apr-YTF intervention, 17 reversed differential metabolic features showed recovery trends.

**Conclusion:**

The active parts of Yangxin Tongmai Formula ameliorated myocardial injury and were associated with partial correction of spatial metabolic remodeling in rats with MIRI.

## 1 Introduction

Myocardial ischemia-reperfusion injury (MIRI) is a major clinical complication after revascularization procedures for acute myocardial infarction and directly affects patients' long-term prognosis (1). Timely reperfusion therapy, including primary percutaneous coronary intervention (PCI) and fibrinolytic therapy, remains the cornerstone strategy for restoring coronary blood flow, salvaging ischemic myocardium, and limiting infarct size in patients with acute myocardial infarction (2). However, the clinical benefit of reperfusion therapy may be limited by delayed treatment initiation, microvascular dysfunction, and the paradoxical injury triggered by the abrupt restoration of blood flow, all of which contribute to MIRI (2). In addition to conventional reperfusion-based treatment, several adjunctive cardioprotective strategies, such as hyperbaric oxygen therapy and hydrogen-based interventions, have been investigated for their potential to attenuate oxidative stress, inflammation, and hypoxia/reoxygenation injury (3, 4). Nevertheless, effective pharmacological interventions for preventing or alleviating MIRI remain limited.

MIRI involves multiple interconnected pathological processes, including oxidative stress, calcium overload, energy- metabolism disturbances, inflammatory responses, and programmed cell death. These processes interact and amplify one another in a cascade, forming a complex pathological network. Because existing interventions often target individual pathways, effectively interrupting this network remains challenging. The active parts of Yangxin Tongmai (apr-YTF) are derived from the classic formula “Yangxin Tongmai” developed by Mr. Qin Bo-wei. apr-YTF comprises the main pharmacologically active components of the original formula and is traditionally used to tonify qi, invigorate blood, and resolve stasis (5). Preclinical studies have shown that apr-YTF improves enzymes involved in myocardial mitochondrial metabolism, alleviates mitochondrial dysfunction related to energy metabolism and transport, and reduces myocardial injury in MIRI model rats with blood stasis syndrome (6, 7).

Matrix-assisted laser desorption/ionization mass spectrometry imaging (MALDI-MSI) is a spatially resolved analytical approach for metabolomics. This label-free, high-resolution technique enables *in situ* visualization and analysis of biomolecules, including lipids and metabolites, within tissue sections, thereby revealing their spatial distribution and metabolic heterogeneity across complex tissues (8). During MIRI, myocardial tissue exhibits marked spatial heterogeneity and regional metabolic remodeling. Conventional homogenate-based metabolomics averages metabolic signals across whole tissue samples and therefore has limited ability to preserve the spatial distribution and dynamic changes of myocardial metabolites (9). In contrast, MALDI-MSI enables *in situ* visualization of metabolite distributions without disrupting tissue architecture, making it suitable for investigating pathological processes such as MIRI that display pronounced regional heterogeneity (10). Previous studies have shown that MALDI-MSI can preserve spatial molecular information within tissue sections and reveal region-dependent metabolic alterations in complex disease tissues (9, 11, 12). Therefore, in the present study, MALDI-MSI was used to characterize the spatial distribution patterns of myocardial metabolites and group-level metabolic remodeling after apr-YTF intervention in rats with MIRI-induced blood stasis syndrome, screen for differential metabolic features and associated pathways, and provide exploratory metabolic evidence for the cardioprotective effects of apr-YTF.

## 2 Materials and methods

### 2.1 Instruments

A fully automated biochemical analyzer (BIOBASE, BK-280), a high-speed refrigerated centrifuge (Hunan Xiangyi Laboratory Instrument Development Co., Ltd., H1650R), a small animal ventilator (Chengdu Taimeng Technology Co., Ltd., HX-101E), a cryostat microtome (Leica, Germany, CM1950), an ultrasound imaging system (Wandong Baisheng Medical Technology Co., Ltd., X5), and a timsTOF fleX mass spectrometry system (Bruker, Germany) were used in this study.

### 2.2 Reagents

Creatine kinase (CK; BIOBASE, batch No. 10426022N), creatine kinase-MB isoenzyme (CK-MB; BIOBASE, batch No. 10413023A), lactate dehydrogenase (LDH; BIOBASE, batch No. 10220020N), and lactate dehydrogenase 1 (LDH1; BIOBASE, batch No. 10405063N) assay kits were used. Triphenyltetrazolium chloride (TTC) staining solution was purchased from Solarbio (batch No. G3005). Hematoxylin and eosin (HE) staining reagent (Solarbio, batch No. G1120) and Masson's trichrome staining reagent (Solarbio, batch No. G1346) were used for histological staining. Isoflurane was obtained from RWD Life Science Co., Ltd. (batch No. 2023110301).

### 2.3 Establishment and validation of the animal model

A total of 55 healthy male SPF-grade Wistar rats (body weight, 200 ± 20 g) were obtained from the Experimental Animal Center of Guangxi University of Chinese Medicine [production license No. SCXK (Xiang) 2024-0023]. After 1 week of acclimatization, 10 rats were allocated to the Control group, and the remaining 45 rats were included in the modeling cohort. Rats in the Control group were maintained under the same housing conditions without left anterior descending artery (LAD) ligation or drug intervention. A MIRI model with blood stasis syndrome was established in the modeling cohort according to the method described by Lei et al. (13). Rats were anesthetized with isoflurane, intubated, and connected to a small animal ventilator. A thoracotomy was performed between the third and fourth left intercostal spaces to expose the heart. The LAD was ligated with 6–0 silk suture for 30 min, after which the ligature was released to allow reperfusion for 2 h. The 2 h reperfusion period represented the acute modeling phase after LAD ligation. Final endpoint measurements were performed after the subsequent 14-day intervention period rather than immediately after the 2 h reperfusion phase. Electrocardiography (ECG) was continuously monitored during the procedure.

Successful MIRI modeling was evaluated using intraoperative ECG changes and model-related validation indicators. Briefly, marked ST-segment elevation with a convex upward curve was observed on ECG after LAD ligation. After reperfusion, serum cardiac enzymes, including CK and LDH, were elevated compared with those in the Control group, and TTC staining showed visible myocardial infarct areas. Rats showing these model-related changes were considered successfully modeled. Blood stasis syndrome-related features were further supported by hemorheological indicators, including whole-blood viscosity at different shear rates, which were used to evaluate the blood stasis phenotype after MIRI modeling.

**Figure 1:**
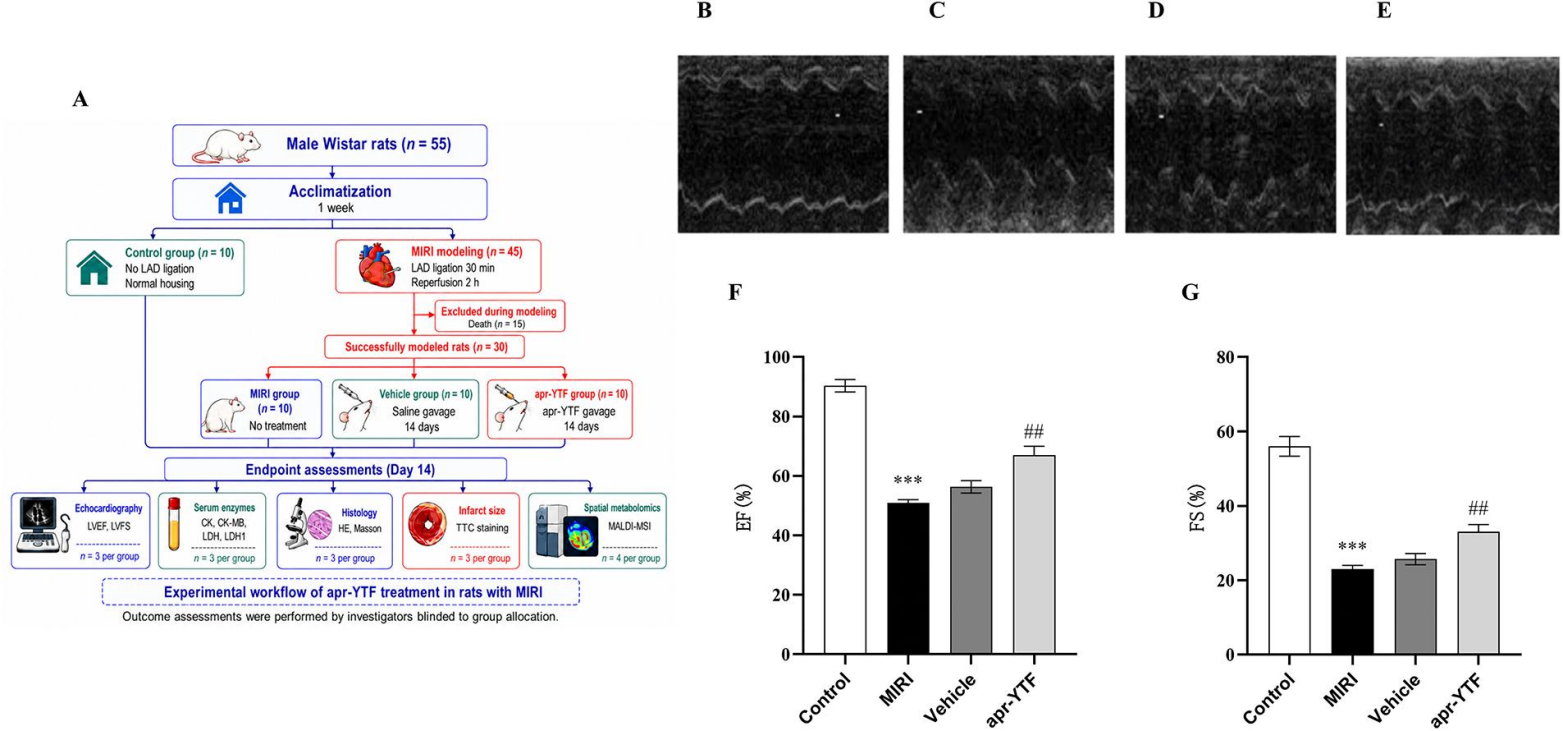
Experimental workflow and effects of apr-YTF on cardiac contractile function in rats with MIRI-induced blood stasis syndrome. **(A)** CONSORT-style animal flow diagram and experimental workflow of apr-YTF treatment in rats with MIRI. A total of 55 male Wistar rats were used. After 1 week of acclimatization, 10 rats were allocated to the Control group, and 45 rats underwent MIRI modeling by LAD ligation for 30 min followed by reperfusion for 2 h. During modeling, 15 rats died, and the successfully modeled rats were randomly assigned to the MIRI, Vehicle, and apr-YTF groups. Endpoint assessments were performed after 14 days of intervention. **(B–E)** Representative echocardiographic images of the Control, MIRI, Vehicle, and apr-YTF groups, respectively. **(F)** Quantitative analysis of left ventricular ejection fraction (LVEF). **(G)** Quantitative analysis of left ventricular fractional shortening (LVFS). Data are presented as mean ± SD, *n* = 3 per group. ***P < 0.001 vs. Control group; ##*P* < 0.01 vs. MIRI group.

During modeling, 15 rats died, and no rats were excluded because of unsuccessful modeling. After successful modeling, the surviving modeled rats (*n* = 30) were randomly assigned to the MIRI, Vehicle, and apr-YTF groups. The final allocated experimental groups were as follows: Control group (*n* = 10), MIRI group (*n* = 10), Vehicle group (*n* = 10), and apr-YTF group (*n* = 10). Rats in the MIRI group were maintained under normal conditions after modeling. Rats in the Vehicle group received 2 mL of saline by gavage to control for gavage and solvent administration. Rats in the apr-YTF group received 2 mL of apr-YTF by gavage once daily, prepared according to the optimized active-fraction combination dose described in the “Experimental Drugs” section. Food and water were provided *ad libitum*, and rats were housed in separate cages for a total intervention period of 2 weeks. The CONSORT-style animal flow diagram is shown in Figure 1A. Outcome assessments were performed by investigators blinded to group allocation. No formal power analysis was performed before the experiment, as acknowledged in the Limitations section.

After the 2-week intervention period, rats were anesthetized by intraperitoneal injection of 2.0% sodium pentobarbital (40–50 mg/kg), and blood samples were collected from the abdominal aorta. Rats were then euthanized by exsanguination under deep anesthesia, and the hearts were rapidly removed. The hearts were washed with saline to remove blood, snap-frozen in liquid nitrogen, and stored at −80°C until further use.

Heart tissues stored at −80°C were equilibrated at −20°C for 1 h in a Leica CM1950 cryostat before being sectioned into 10 μm-thick slices. The sections were transferred to pre-chilled indium tin oxide (ITO) slides, thaw-mounted onto the slides, and allowed to dry at room temperature to remove residual moisture. The sections were then vacuum-dried for 30 min.

The animal experiment was approved by the Animal Welfare and Ethics Committee of Guangxi University of Chinese Medicine (DW20220408-042), and all animal procedures complied with ethical principles for the use of laboratory animals.

### 2.4 Experimental drugs

The Yangxin Tongmai Active Constituents Formula (apr-YTF) is a standardized preparation composed of the main active fractions of the original Yangxin Tongmai Formula, combined in optimized proportions. According to a previous study (14), the original Yangxin Tongmai Formula consisted of Panax ginseng C.A. Mey. 20 g, Cinnamomi Ramulus 15 g, Rehmanniae Radix 15 g, Salvia miltiorrhiza Bunge 25 g, and Alismatis Rhizoma 10 g. apr-YTF consists of ginsenosides, tanshinone IIA, ginseng polysaccharides, total alkaloids, and the total volatile oil fraction.

Each active fraction was extracted and purified from the herbal materials of the original Yangxin Tongmai Formula, which were provided by the Traditional Chinese Medicine Pharmacy of the Affiliated Hospital of Guangxi University of Chinese Medicine and identified as conforming to the standards of the Chinese Pharmacopoeia. The preparation methods had been previously established by the research team: ginsenosides were purified using D101 macroporous resin, tanshinone IIA was purified by silica gel column chromatography; ginseng polysaccharides were obtained by water extraction followed by ethanol precipitation; total alkaloids were prepared by acid extraction followed by alkaline precipitation; and the total volatile oil fraction was obtained by steam distillation.

According to the previous study (14), the active fractions were separated and purified, with a reported purity of more than 70%. The optimized active-fraction combination dose was as follows: ginsenosides, 10.3 g·kg^−1^ body weight; tanshinone IIA, 0.38 g·kg^−1^ body weight; ginseng polysaccharides, 1.43 g·kg^−1^ body weight; total alkaloids, 0.32 g·kg^−1^ body weight; and total volatile oil fraction, 18.65 mL·kg^−1^ body weight. This active-fraction combination dose was selected based on the previously reported optimized compatibility of Yangxin Tongmai Formula for anti-acute myocardial ischemia effects (14). Therefore, this established optimized combination was used in the present study to investigate the effects of apr-YTF on myocardial injury and spatial metabolic remodeling in the MIRI model. The total volatile oil fraction was emulsified with Tween before use, and the preparation was diluted with distilled water to the required concentration immediately before administration. All apr-YTF preparations used in the present study were prepared from the same batch to minimize batch-related variability.

### 2.5 Echocardiographic assessment of cardiac contractile function

Fourteen days after induction of the MIRI-induced blood stasis syndrome, echocardiographic examinations were performed in three rats randomly selected from each group to assess cardiac contractile function. A small animal ultrasound system was used to record echocardiograms for each rat. Two-dimensional (B-mode) and M-mode ultrasound images were acquired at the level of the left ventricular papillary muscles in the short-axis view. Three complete cardiac cycles were recorded continuously, and the average values were calculated. The measured parameters included left ventricular ejection fraction (LVEF) and left ventricular fractional shortening (LVFS).

### 2.6 Biochemical analysis to assess the degree of myocardial injury

Blood samples were collected from three randomly selected rats in each group via the abdominal aorta at the time of tissue harvesting and centrifuged at 2,500 rpm and 4°C for 10 min. The supernatant was collected, and serum levels of CK, CK-MB, LDH, and LDH1 were measured using an automated biochemical analyzer and the corresponding reagent kits to assess myocardial injury.

### 2.7 Observation of myocardial tissue pathological structure via HE staining

Myocardial tissues from three randomly selected rats in each group were fixed in 4% paraformaldehyde, embedded in paraffin, and sectioned at 4–5 μm. Sections were stained with HE and examined under a light microscope to evaluate myocardial structure and inflammatory infiltration. HE staining and Masson staining were performed using adjacent myocardial tissue sections from the same three randomly selected rats in each group.

### 2.8 Quantitative assessment of myocardial fibrosis in rat myocardial tissue via masson staining

Adjacent myocardial tissue sections from the same three rats used for HE staining in each group were stained with Masson's trichrome. Collagen fibers appeared blue, whereas cardiomyocytes appeared red. The percentage of collagen area was calculated using ImageJ as follows: collagen area (%) = (collagen area/total myocardial area) × 100%.

### 2.9 Quantitative assessment of myocardial infarction area in rat myocardial tissue via TTC staining

Frozen cardiac sections from three rats in each group were stained with TTC. Viable myocardium appeared red, whereas infarcted tissue appeared white. ImageJ was used to calculate the infarcted area percentage as follows: infarcted area (%) = infarcted area/total area × 100%.

### 2.10 MALDI-MSI analysis

#### 2.10.1 Sample preparation

Frozen cardiac tissue was sectioned into 10 μm-thick sections and mounted onto ITO-coated glass slides. A 15 mg/mL 2,5-dihydroxybenzoic acid (DHB) matrix solution was prepared in acetonitrile/water (9:1, v/v) and uniformly sprayed onto the tissue sections. DHB was selected because it is one of the most widely used matrices in MALDI-MSI and provides broad coverage for small metabolites and many lipid species in tissue sections (15). Positive-ion mode was used for untargeted spatial metabolomics because it generally enables broad detection of lipid-related and small-molecule metabolic features under the present experimental conditions. Negative-ion mode and alternative matrices, such as 9-aminoacridine (9-AA), were not used in this study.

#### 2.10.2 Mass spectrometry imaging and structural identification

A Bruker trapped ion mobility spectrometry-time-of-flight (TIMS-TOF) flex mass spectrometer was used for imaging in positive-ion mode, with a spatial resolution of 50 μm and a mass range of 50–1,300 Da. Data were imported into SCiLS Lab software for processing. Metabolite annotation was performed based on accurate precursor mass, on-tissue tandem mass spectrometry (MS/MS) spectra, and database matching. For high-intensity ions, on-tissue MS/MS spectra were acquired directly from tissue sections and matched against an in-house database integrated with public metabolite databases, including the Human Metabolome Database (HMDB). Metabolites supported by MS/MS spectral matching were regarded as MS/MS-supported putative annotations, whereas features annotated only by accurate mass matching were considered putatively annotated metabolites. Because authentic standard-based validation and extract-based targeted liquid chromatography-tandem mass spectrometry (LC-MS/MS) confirmation were not performed, these annotations were not assigned as level-1 confirmed identifications.

### 2.11 Data processing and analysis

The MSI data were imported into SCiLS Lab software for preprocessing, normalization, visualization, and statistical analysis. Root mean square (RMS) normalization was applied to reduce spectrum-to-spectrum signal intensity variation across tissue sections. Mass accuracy was monitored during data acquisition, and lock-mass correction was applied when mass drift was observed. All tissue sections were processed under consistent matrix-deposition conditions and acquired using the same instrumental parameters to minimize technical variability. Quantitative data are presented as mean ± SD. Comparisons among multiple groups were performed using one-way analysis of variance (ANOVA) followed by the least significant difference (LSD) test (*P* < 0.05). For spatial metabolomics, independent-samples t-tests and false discovery rate (FDR) correction were employed to screen for differentially expressed metabolites (*P* < 0.05).

## 3 Results

### 3.1 Effects of apr-YTF on cardiac function in rats with MIRI-induced blood stasis syndrome

Echocardiographic results (Figures 1B–G) showed that 14 days after induction of MIRI-induced blood stasis syndrome, cardiac function, particularly systolic function, was significantly impaired, as reflected by reduced LVEF and LVFS. In contrast, apr-YTF significantly alleviated MIRI-induced cardiac dysfunction, with improvements observed in all relevant cardiac function parameters (*P* < 0.001).

### 3.2 Effects of apr-YTF on serum cardiac enzyme levels in rats with MIRI-induced blood stasis syndrome

**Figure 2:**
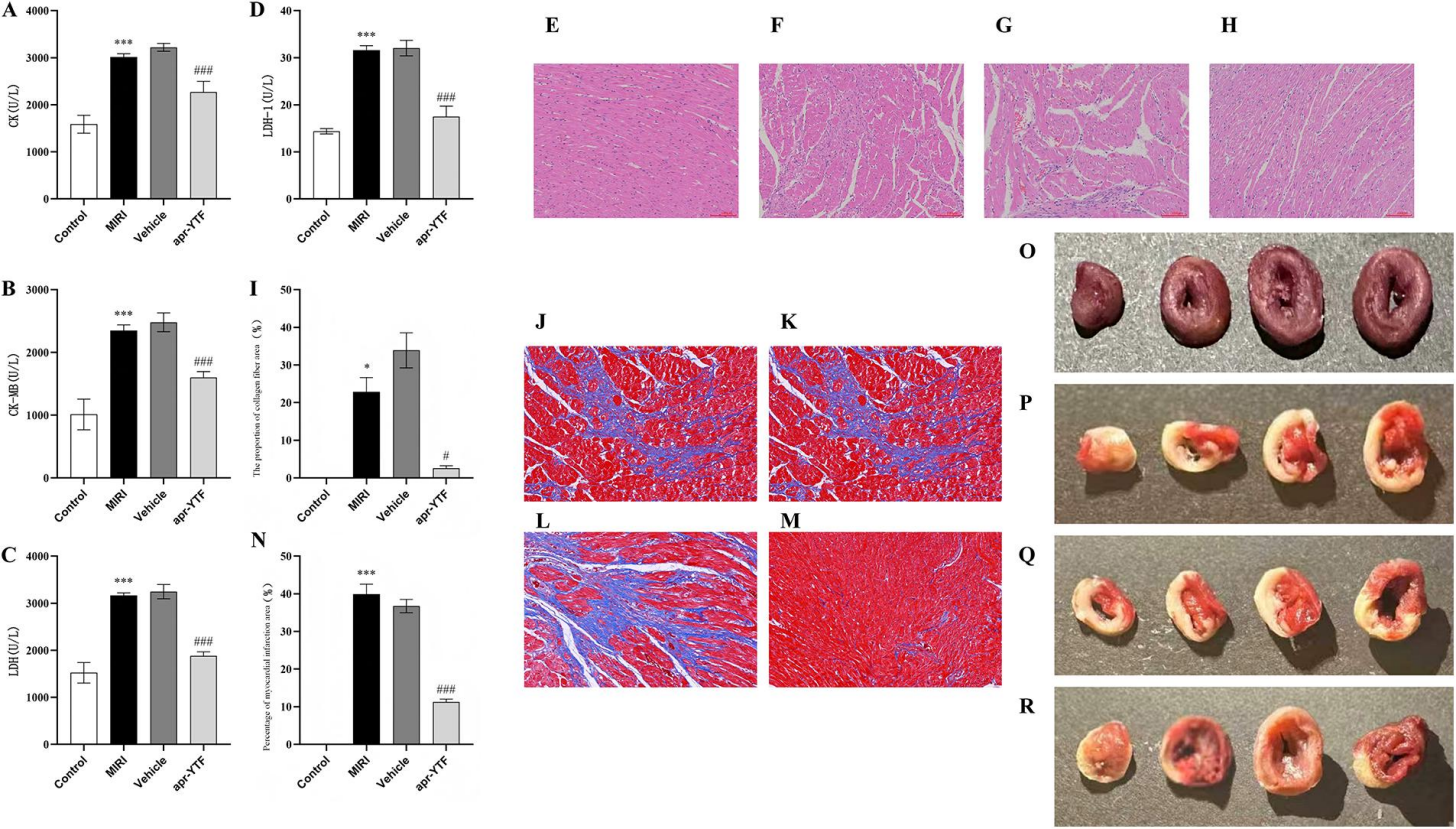
Effects of apr-YTF on myocardial injury, histopathological changes, myocardial fibrosis, and infarct size in rats with MIRI-induced blood stasis syndrome. **(A–D)** Serum levels of myocardial injury-related enzymes, including creatine kinase (CK), creatine kinase-MB isoenzyme (CK-MB), lactate dehydrogenase (LDH), and lactate dehydrogenase 1 (LDH1). **(E–H)** Representative hematoxylin and eosin (HE)-stained myocardial sections from the Control, MIRI, Vehicle, and apr-YTF groups, respectively. **(I)** Quantitative analysis of myocardial collagen fiber area. **(J–M)** Representative Masson's trichrome-stained myocardial sections from the Control, MIRI, Vehicle, and apr-YTF groups, respectively. Collagen fibers are stained blue, and cardiomyocytes are stained red. **(N–Q)** Representative triphenyltetrazolium chloride (TTC)-stained myocardial sections from the Control, MIRI, Vehicle, and apr-YTF groups, respectively. Red areas indicate viable myocardium, whereas white areas indicate infarcted myocardium. **(R)** Quantitative analysis of myocardial infarct area. Data are presented as mean ± SD, *n* = 3 per group. *P < 0.05, ***P < 0.001 vs. Control group; #*P* < 0.05, ###*P* < 0.001 vs. MIRI group.

Serum concentrations of CK, CK-MB, LDH, and LDH1 were measured in each group using commercial kits. As shown in Figures 2A–D, serum cardiac injury markers were significantly higher in the MIRI group than in the Control group (*P* < 0.001). apr-YTF significantly reduced the elevated serum levels of CK, CK-MB, LDH, and LDH1 (*P* < 0.001).

### 3.3 Effects of apr-YTF on the pathological morphology of myocardial tissue in rats with MIRI-induced blood stasis syndrome

HE staining (Figures 2E–H) showed that myocardial tissue in the Control group had intact architecture, regularly arranged cardiomyocytes, and uniform cytoplasmic staining. Only a few inflammatory cells were scattered within the stroma, and no fibrous tissue proliferation or hemorrhagic changes were observed. In the MIRI group, myocardial tissue exhibited obvious structural injury, including disorganized cardiomyocyte arrangement, extensive myofibrillar dissolution and rupture, markedly widened myocardial stroma, and edema. The Vehicle group also showed injury, including myocardial fiber rupture and interstitial edema. In the apr-YTF group, myocardial structural damage was markedly attenuated, with more orderly cardiomyocyte arrangement, relatively uniform cytoplasmic staining, and no obvious inflammatory cell infiltration.

### 3.4 Effects of apr-YTF on myocardial fibrosis in rats with MIRI-induced blood stasis syndrome

Masson's staining (Figures 2I–M) showed that myocardial tissue in the Control group was predominantly red, with neatly arranged myocardial fibers and only a small amount of collagen fibers in the interstitium. In the MIRI and Vehicle groups, diffuse collagen fiber proliferation was observed in the myocardial stroma and perivascular spaces. These collagen fibers appeared as blue-stained bundles or networks with disorganized arrangement and were accompanied by inflammatory cell infiltration, indicating significant myocardial fibrosis (*P* < 0.05). In the apr-YTF group, collagen deposition was significantly reduced (*P* < 0.05), as shown by decreased blue-stained area, lower collagen volume fraction, and a more regular myocardial fiber structure.

### 3.5 Effect of apr-YTF on the area of myocardial infarction in rats with MIRI and blood stasis syndrome

TTC staining showed that viable myocardium appeared red, whereas infarcted areas appeared white. Both the MIRI and Vehicle groups exhibited extensive white infarcted areas in myocardial tissue, with a significant increase in infarct area (*P* < 0.001). In contrast, the infarct area was significantly reduced in the apr-YTF group (*P* < 0.001), suggesting that apr-YTF attenuated ischemia-reperfusion-induced myocardial infarction (Figures 2N–R).

### 3.6 Effects of apr-YTF on the spatial metabolic profile of myocardial tissue in rats with MIRI-induced blood stasis syndrome

#### 3.6.1 Spatial distribution of myocardial metabolic features

**Figure 3:**
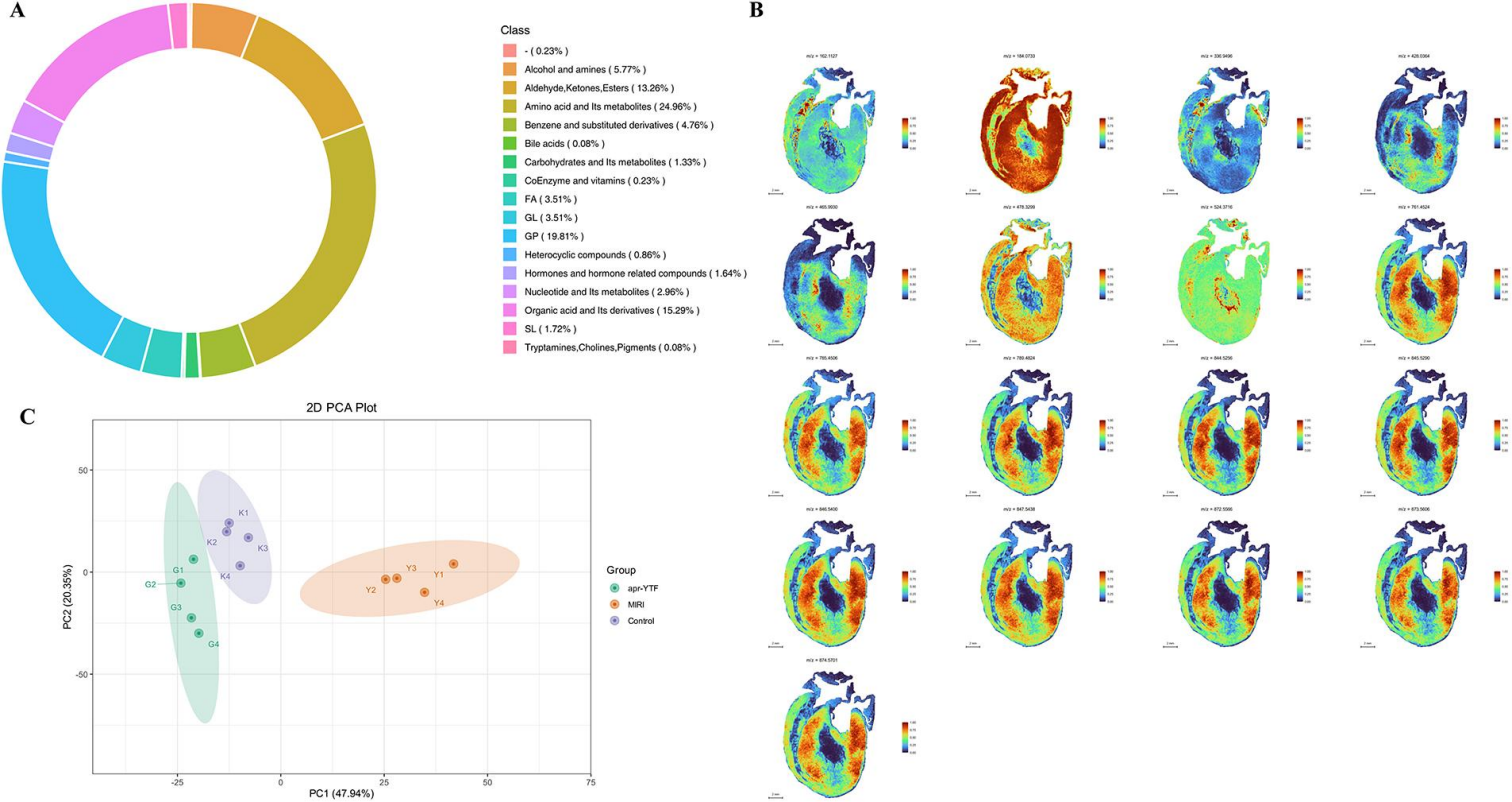
MALDI-MSI-based spatial metabolic profiling of myocardial tissues in rats with MIRI-induced blood stasis syndrome. **(A)** Classification distribution of metabolites detected by matrix-assisted laser desorption/ionization mass spectrometry imaging (MALDI-MSI) in positive-ion mode. **(B)** Representative ion images showing the spatial distribution of selected metabolic features in rat myocardial tissue. The color scale indicates relative ion intensity from low to high. **(C)** Principal component analysis (PCA) score plot of myocardial metabolic profiles among the Control, MIRI, and apr-YTF groups. Each point represents one biological sample, and the ellipse indicates the distribution tendency of each group. MALDI-MSI analysis was performed using myocardial tissues from 4 rats per group.

A total of 1,282 metabolites were identified in the positive-ion mode by MALDI-MSI, of which 468 were supported by on-tissue MS/MS spectra. The major categories were amino acids (320), glycerophospholipids (254), and organic acids (196) (Figure 3A). Metabolites displayed heterogeneous spatial distributions across myocardial sections (Figure 3B): adenosine diphosphate (ADP) and guanosine diphosphate (GDP) were mainly enriched in the left ventricle and apical region; phosphoenolpyruvate (PEP) and L-carnitine were concentrated around the right ventricle; and glycerophospholipids containing polyunsaturated fatty acid chains, such as PE-NMe(20:0/22:6), were primarily distributed in the walls of the left and right ventricles.

#### 3.6.2 Analysis of differential metabolites

Principal component analysis (PCA) showed that the metabolic profiles of the Control and MIRI groups were clearly separated, indicating marked changes in myocardial metabolism after MIRI modeling. The apr-YTF group was positioned between the Control and MIRI groups and closer to the Control group, suggesting that apr-YTF intervention shifted the metabolic profile toward that of the Control group (Figure 3C).

#### 3.6.3 Construction and validation of multivariate statistical analysis models

**Figure 4:**
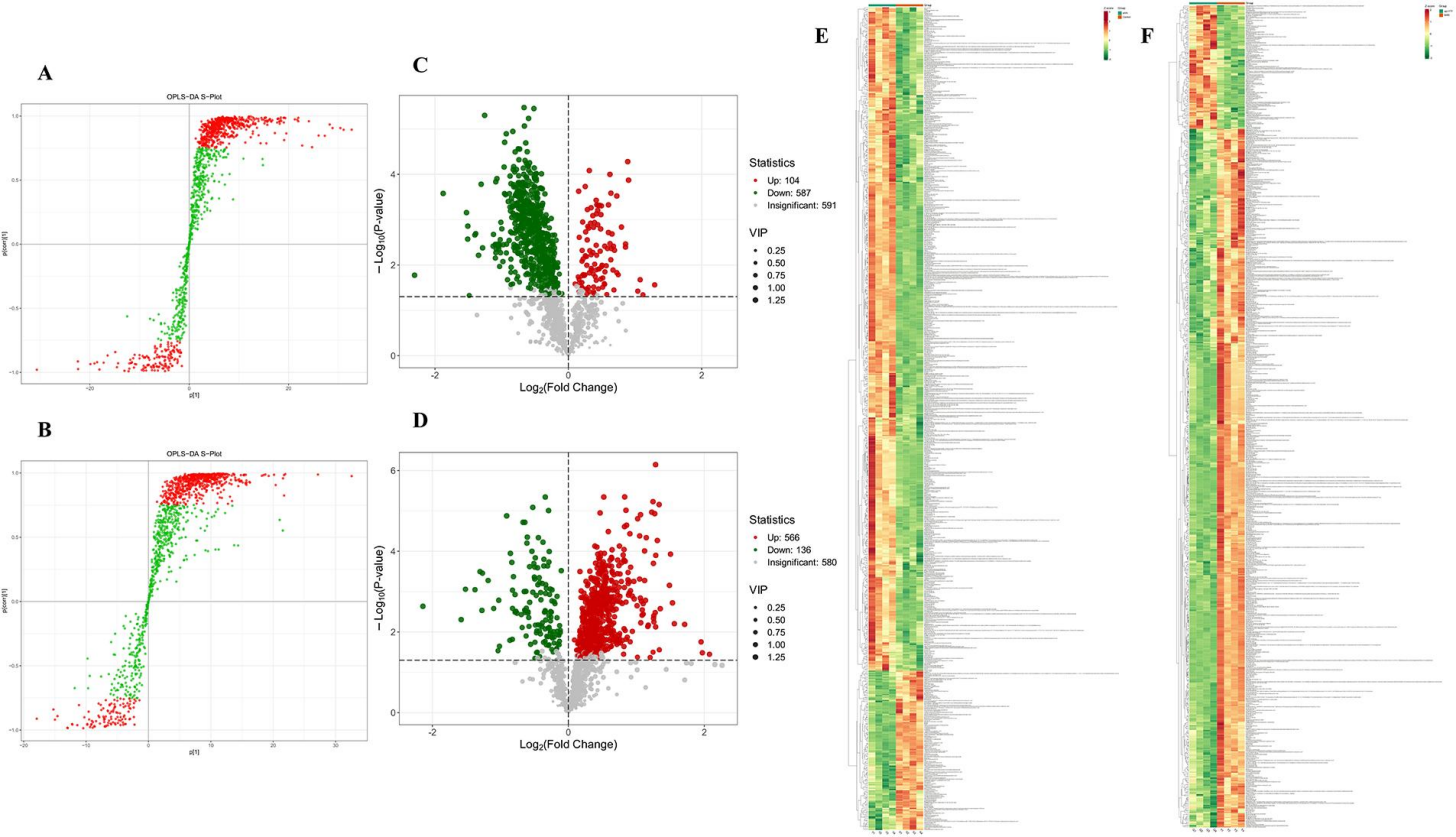
Multivariate and univariate analyses of differential metabolic features in myocardial tissues. **(A,C,E)** S-plot, volcano plot, and hierarchical clustering heatmap for the comparison between the apr-YTF group and the MIRI group. **(B,D,F)** S-plot, volcano plot, and hierarchical clustering heatmap for the comparison between the MIRI group and the Control group. In the S-plots, red points represent metabolic features with variable importance in projection (VIP) > 1. In the volcano plots, red and green points represent upregulated and downregulated differential metabolic features, respectively, whereas gray points represent features that did not meet the differential screening criteria. In the heatmaps, red indicates relatively high abundance and green indicates relatively low abundance. Differential metabolic features were screened based on fold change, *P* value, and VIP value.

In the S-plot, the red data points represent metabolites with variable importance in projection (VIP) > 1. A large number of differential metabolic features were observed between the comparison groups (Figures 4A,B).

#### 3.6.4 Identification of differentially expressed metabolites using multivariate statistical analysis models

Using VIP > 1 and *P* < 0.05 as screening criteria, 702 differential metabolic features (566 upregulated and 136 downregulated) were identified in the MIRI group compared with the Control group. These features mainly included amino acids, L-carnitine, creatinine, glycerophospholipids, and adenosine monophosphate (AMP). A comparison between the apr-YTF and MIRI groups identified 691 differential metabolic features (104 upregulated and 587 downregulated), with many features showing a tendency to shift toward levels observed in the Control group after apr-YTF intervention (Figures 4C,D).

#### 3.6.5 Hierarchical cluster analysis

Hierarchical cluster analysis (HCA) was performed on the differential metabolic features. Samples from the MIRI and Control groups formed distinct clusters, with good intra-group consistency. In the MIRI group, elevated metabolites were mainly glycerophospholipids and acylcarnitines, whereas decreased metabolites were mainly nucleotides and coenzymes. After apr-YTF intervention, metabolite expression patterns shifted toward those of the Control group (Figures 4E,F).

#### 3.6.6 Metabolic pathway enrichment analysis

**Figure 5:**
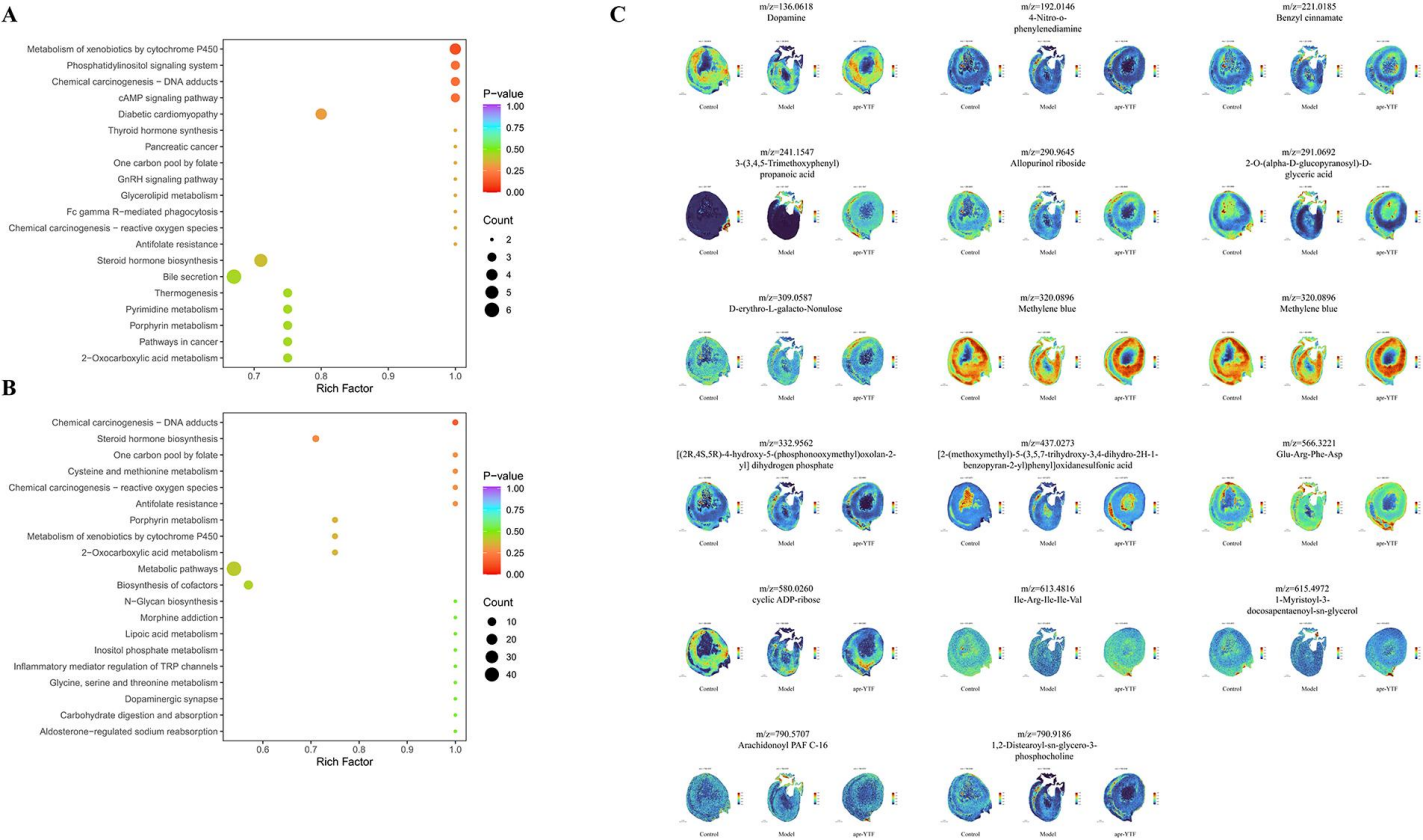
Pathway enrichment analysis and spatial distribution of reversed differential metabolic features after apr-YTF intervention. **(A)** Metabolic pathway enrichment analysis of differential metabolic features between the MIRI group and the Control group. **(B)** Metabolic pathway enrichment analysis of differential metabolic features between the apr-YTF group and the MIRI group. The bubble size represents the number of enriched metabolic features, and the color indicates the *P* value. **(C)** Spatial ion images of 17 reversed differential metabolic features in myocardial tissues from the Control, MIRI, and apr-YTF groups. These features showed marked changes after MIRI modeling and an opposite or recovery trend after apr-YTF intervention. The statistical and annotation information for the 17 reversed differential metabolic features, including fold change, VIP values, *P* values, FDR-adjusted q values, MS/MS support status, and annotation confidence, is summarized in Table 2. The color scale indicates relative ion intensity from low to high.

Differential metabolic features were annotated against the Kyoto Encyclopedia of Genes and Genomes (KEGG) database for metabolic pathway enrichment analysis (MSEA). In the MIRI group, these features were primarily enriched in purine metabolism, amino acid metabolism (tryptophan, tyrosine, and arginine), and cofactor biosynthesis pathways. In the apr-YTF group, differential metabolic features were enriched in pathways including metabolic networks, cofactor biosynthesis, glycerophospholipid metabolism, purine metabolism, and sphingolipid metabolism (Figures 5A,B).

Topological analysis of metabolic pathways (with FC > 1.5 or <0.67, *P* < 0.05, VIP > 1) identified 13 MIRI model-associated pathways (Table 1). Pathways with higher impact values included phenylalanine/tyrosine/tryptophan biosynthesis (0.50), phenylalanine metabolism (0.36), tyrosine metabolism (0.13), and glycerophospholipid metabolism (0.11).

**Table 1:** MIRI model-associated differential metabolic pathways.

Pathway	Total	Expected	Hits	Raw p	#NAME	Holm adjust	FDR	Impact
One carbon pool by folate	26	0.14698	2	0.0086086	2.0651	0.68869	0.55967	0.06844
Glycerophospholipid metabolism	36	0.20352	2	0.016203	1.7904	1	0.55967	0.11201
Phenylalanine, tyrosine and tryptophan biosynthesis	4	0.022613	1	0.022443	1.6489	1	0.55967	0.5
Linoleic acid metabolism	5	0.028266	1	0.027983	1.5531	1	0.55967	0
Phenylalanine metabolism	8	0.045226	1	0.044437	1.3523	1	0.71099	0.35714
alpha-Linolenic acid metabolism	13	0.073492	1	0.071311	1.1468	1	0.87547	0
Arginine biosynthesis	14	0.079146	1	0.076604	1.1157	1	0.87547	0
Sphingolipid metabolism	32	0.1809	1	0.16741	0.77622	1	1	0
Cysteine and methionine metabolism	33	0.18656	1	0.17221	0.76394	1	1	0.05271
Arginine and proline metabolism	36	0.20352	1	0.18648	0.72938	1	1	0
Tyrosine metabolism	42	0.23744	1	0.21435	0.66888	1	1	0.12972
Arachidonic acid metabolism	44	0.24874	1	0.22345	0.65082	1	1	0
Steroid hormone biosynthesis	87	0.49183	1	0.39776	0.40038	1	1	0

#### 3.6.7 Reversal of MIRI-associated metabolic differences by apr-YTF

**Figure 6:**
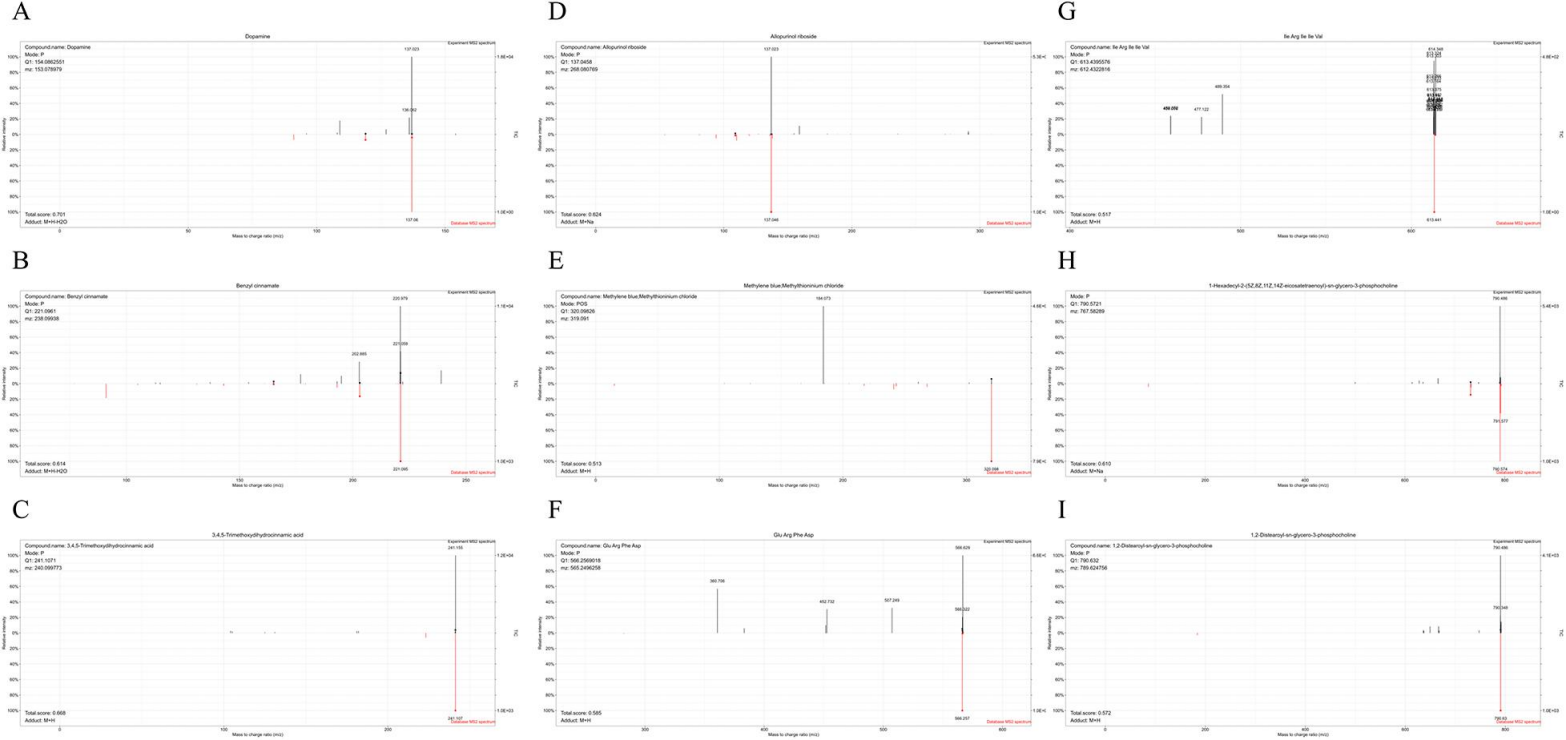
On-tissue MS/MS spectra of nine representative reversed differential metabolic features with MS/MS support. The MS/MS spectra are shown for nine reversed differential metabolic features that were improved after apr-YTF intervention and supported by on-tissue tandem mass spectrometry (MS/MS) spectral matching: **(A)** dopamine, m/z 136.0618; **(B)** benzyl cinnamate, m/z 221.0185; **(C)** 3-(3,4,5-trimethoxyphenyl)propanoic acid, m/z 241.1547; **(D)** 2-O-(α-D-glucopyranosyl)-D-glyceric acid, m/z 291.0692; **(E)** methylene blue, m/z 320.0896; **(F)** Glu-Arg-Phe-Asp, m/z 566.3221; **(G)** Ile-Arg-Ile-Ile-Val, m/z 613.4816; **(H)** arachidonoyl platelet-activating factor C-16, m/z 790.4860; and **(I)** 1,2-distearoyl-sn-glycero-3-phosphocholine, m/z 790.5350.

After the top differential metabolic features in the MIRI group were screened based on the magnitude of upregulation and downregulation, 17 features showed recovery trends following apr-YTF intervention. Among these reversed metabolic features, several representative features were putatively annotated as cyclic adenosine diphosphate ribose (cADPR), dopamine, distearoylphosphatidylcholine (DSPC), and allopurinol riboside. These features were mainly associated with glycerophospholipid metabolism and purine-related metabolic remodeling. Notably, the feature putatively annotated as cADPR may provide a metabolic clue related to calcium-homeostasis regulation; however, this interpretation remains exploratory and requires further validation by functional calcium-handling assays (Figure 5C). The statistical and annotation information for the 17 reversed differential metabolic features, including fold change, VIP values, *P* values, FDR-adjusted q values, MS/MS support status, and annotation confidence, is summarized in Table 2. Among these features, nine were supported by on-tissue MS/MS spectra, as shown in Figure 6.

**Table 2:** Quantitative statistical and annotation information of the 17 reversed differential metabolic features after apr-YTF intervention.

Name	Formula	Class I	Precursor (Da)	*P*-value (MIRI/Control)	*P*-value (apr-YTF/MIRI)	VIP (MIRI/Control)	VIP (apr-YTF/MIRI)	FDR/ q-value (MIRI/Control)	FDR/ q-value (apr-YTF/MIRI)	FC with direction (MIRI/Control)	FC with direction (apr-YTF/MIRI)	MS/MS support	Annotation confidence
3-(3,4,5-Trimethoxyphenyl)propanoic acid	C12H16O5	Organic acid and Its derivatives	241.1547	0.017592131	0.016789918	1.241760144	1.296494401	0.038618342	0.03654444	3.87↑	0.20↓	Yes	MS/MS-supported putative annotation
1,2-Distearoyl-sn-glycero-3-phosphocholine	C44H88NO8P	GP	790.9186	0.001901775	0.001386638	1.31090491	1.307376357	0.009752303	0.006802271	2.89↑	0.35↓	Yes	MS/MS-supported putative annotation
4-Nitro-o-phenylenediamine	C6H7N3O2	Benzene and substituted derivatives	192.0146	0.000118072	0.000699464	1.289949991	1.317662614	0.00369842	0.005062466	2.84↑	0.35↓	No	Putative annotation based on accurate mass and database matching
2-O-(alpha-D-glucopyranosyl)-D-glyceric acid	C9H16O9	Organic acid and Its derivatives	291.0692	0.000700443	0.00259406	1.294497852	1.171388754	0.00636857	0.009752449	2.80↑	0.59↓	Yes	MS/MS-supported putative annotation
Glu-Arg-Phe-Asp	C24H35N7O9	Amino acid and Its metabolites	566.3221	0.002656854	0.004320909	1.307543783	1.228163502	0.011546057	0.013544171	2.55↑	0.59↓	Yes	MS/MS-supported putative annotation
Allopurinol riboside	C10H12N4O5	Nucleotide and Its metabolites	290.9645	0.00000697454604875885	0.0000127564291736061	1.311749266	1.302414472	0.0012155524045447	0.002336249	2.52↑	0.52↓	No	Putative annotation based on accurate mass and database matching
Arachidonoyl PAF C-16	C44H82NO7P	GP	790.5707	0.0000542149630581803	0.001543676	1.311209806	1.19476327	0.002673215	0.007196335	2.48↑	0.58↓	Yes	MS/MS-supported putative annotation
[(2R,4S,5R)-4-hydroxy-5-(phosphonooxymethyl)oxolan-2-yl] dihydrogen phosphate	C5H12O10P2	Organic acid and Its derivatives	332.9562	0.000126115	0.000165299	1.262267824	1.313709673	0.003759994	0.003737975	2.40↑	0.40↓	No	Putative annotation based on accurate mass and database matching
Benzyl cinnamate	C16H14O2	Benzene and substituted derivatives	221.0185	0.007509556	0.010766258	1.249920494	1.193207385	0.022337009	0.0261408	2.44↑	0.49↓	Yes	MS/MS-supported putative annotation
D-erythro-L-galacto-Nonulose	C9H18O9	Carbohydrates and Its metabolites	309.0587	0.005630618	0.023850823	1.262020512	1.018656575	0.018091359	0.048689099	2.39↑	0.60↓	No	Putative annotation based on accurate mass and database matching
cyclic ADP-ribose	C15H21N5O13P2	Carbohydrates and Its metabolites	580.026	0.002310766	0.045373106	1.30219249	1.116710178	0.010622107	0.08093958	0.22↓	2.17↑	No	Putative annotation based on accurate mass and database matching
Dopamine	C8H11NO2	Alcohol and amines	136.0618	0.000320264	0.016434484	1.237040238	1.071303725	0.004677994	0.035892689	0.41↓	1.68↑	Yes	MS/MS-supported putative annotation
[2-(methoxymethyl)-5-(3,5,7-trihydroxy-3,4-dihydro-2H-1-benzopyran-2-yl)phenyl]oxidanesulfonic acid	C17H18O9S	Organic acid and Its derivatives	437.0273	0.002918988	0.021129024	1.299788425	1.221960788	0.012189391	0.044025483	0.41↓	2.11↑	No	Putative annotation based on accurate mass and database matching
Methylene blue	C16H18ClN3S	Heterocyclic compounds	320.0896	0.000013274363232158	0.000134413	1.313133425	1.30875804	0.001215552	0.003737975	0.53↓	1.87↑	Yes	MS/MS-supported putative annotation
Jnj-42041935	C12H6ClF3N4O3	Organic acid and Its derivatives	329.0052	0.000112885	0.004707989	1.317744705	1.273761213	0.00369842	0.014235004	0.55↓	1.61↑	No	Putative annotation based on accurate mass and database matching
Ile-Arg-Ile-Ile-Val	C29H56N8O6	Amino acid and Its metabolites	613.4816	0.000188925	5.24593E-05	1.291400127	1.305881353	0.004100864	0.003673775	0.56↓	1.85↑	Yes	MS/MS-supported putative annotation
1-Myristoyl-3-docosapentaenoyl-sn-glycerol	C39H66O5	GL	615.4972	0.003279403	0.003851405	1.261451606	1.266768783	0.012663238	0.012563615	0.64↓	1.60↑	No	Putative annotation based on accurate mass and database matching

“MS/MS-supported putative annotation” indicates that the feature was annotated based on accurate precursor mass and on-tissue MS/MS spectral matching with metabolite databases. “Putative annotation based on accurate mass and database matching” indicates that the feature was annotated mainly according to accurate precursor mass and database-assisted matching. Authentic standard-based validation and extract-based targeted LC-MS/MS confirmation were not performed; therefore, these features were not assigned as level-1 confirmed identifications.

## 4 Discussion

The pathological basis of MIRI involves a complex network of energy-metabolism disturbance, oxidative stress, calcium overload, mitochondrial dysfunction, inflammatory responses, and programmed cell death (16–18). Conventional homogenate-based metabolomics averages metabolic signals across whole-tissue samples and therefore has limited ability to preserve the spatial distribution and regional metabolic heterogeneity of myocardial tissue (9, 12). Using MALDI-MSI, this study characterized the *in situ* spatial distribution of myocardial metabolites and explored metabolic remodeling associated with apr-YTF intervention in rats with MIRI-induced blood stasis syndrome. apr-YTF significantly improved cardiac function, reduced myocardial injury, decreased infarct size, and alleviated fibrosis and inflammation (19). Spatial metabolomics suggested that these protective effects may be associated with coordinated remodeling of energy, lipid, and amino acid metabolic networks (20).

### 4.1 Metabolic compartments in the normal heart and metabolic reprogramming in MIRI

MALDI-MSI revealed that metabolite distribution in the normal heart was closely aligned with functional compartments: ADP/GDP was enriched in the left ventricle and apical region, consistent with high energy demand; polyunsaturated fatty acid-containing phospholipids were concentrated in the ventricular walls, supporting mitochondrial membrane function (21); and PEP and L-carnitine were enriched around the right ventricle. These data provide a spatial map of metabolic organization in the normal rat heart.

Under MIRI conditions, L-carnitine was significantly upregulated, suggesting altered fatty-acid β-oxidation (22, 23). Elevated creatinine reflected depletion of high-energy phosphates (24, 25), whereas decreased AMP indicated an imbalance in energy charge (26). Glycerophospholipid metabolites were generally upregulated, suggesting membrane phospholipid remodeling (27, 28). Together, these changes suggest a pathological cascade involving energy-metabolism dysfunction, membrane structural damage, and mitochondrial dysfunction.

### 4.2 Multipathway regulatory roles of apr-YTF

MIRI involves the interplay of multiple pathways. Among them, glycerophospholipid metabolism showed a prominent impact, as its products can activate inflammation and apoptosis (29, 30); the sphingolipid metabolite ceramide induces mitochondrial apoptosis (31); disruptions in phenylalanine/tyrosine metabolism affect catecholamine synthesis (32); and disruptions in one-carbon metabolism affect myocardial repair (33). Unlike previous studies that primarily focused on single pathways (2), the present study found that MIRI involves synergistic disruptions across multiple pathways, forming a complex pathological network.

After apr-YTF intervention, differential metabolic features were enriched in pathways related to metabolic networks, cofactor synthesis, glycerophospholipid metabolism, purine metabolism, and sphingolipid metabolism, suggesting multi-pathway synergistic regulation. This finding aligns with the holistic action characteristics of traditional Chinese herbal formulas (34) and is consistent with previous studies showing that Yangxin Tongmai Formula improves mitochondrial function (7).

### 4.3 Potential biological implications of reversed metabolic features

Among the 17 metabolic features reversed by apr-YTF, the change in the feature putatively annotated as cADPR was noteworthy. cADPR has been reported to participate in intracellular calcium mobilization through ryanodine receptor-related signaling (35). During ischemia-reperfusion, disturbed calcium homeostasis may promote mitochondrial calcium overload, mitochondrial permeability transition pore opening, and subsequent cell injury. In the present study, the metabolic feature putatively annotated as cADPR showed a reversed trend after apr-YTF intervention, suggesting a possible association between apr-YTF-mediated metabolic remodeling and calcium-homeostasis-related regulation. However, this finding was derived from spatial metabolomic annotation and does not directly demonstrate activation or inhibition of the cADPR/Ca^2+^ signaling axis. Because RyR2 expression or activity, CD38 activity, Ca^2+^ transients, mitochondrial Ca^2+^ accumulation, and mitochondrial permeability transition pore opening were not examined, the involvement of cADPR/Ca^2+^-related mechanism handling should be regarded as a hypothesis-generating observation rather than a confirmed mechanism. Lee et al. (36) previously reported the protective role of cADPR-related signaling in myocardial ischemia.

The feature putatively annotated as allopurinol riboside may reflect purine-related metabolic remodeling after MIRI and apr-YTF intervention. Importantly, this feature was not annotated as free allopurinol. Because isomeric ambiguity may exist in untargeted metabolomics, especially among purine nucleoside-related features, this annotation should be interpreted cautiously. The rebound in dopamine levels suggests the possible involvement of neuro-immune regulation, as dopamine can activate antioxidant pathways via its receptors (37). The decrease in oxidized glutathione further supports the potential antioxidant effects of apr-YTF (38, 39).

### 4.4 The unique value of spatial metabolomics

Unlike traditional tissue homogenate-based metabolomics, MALDI-MSI preserves spatial distribution information and enables visualization of metabolite heterogeneity across myocardial tissue sections (10, 40, 41). In the present study, MALDI-MSI revealed whole-section spatial distribution patterns of myocardial metabolites after MIRI and apr-YTF intervention, providing an *in situ* perspective for understanding myocardial metabolic remodeling. Spatial ion images of representative reversed metabolic features, including putatively annotated cyclic adenosine diphosphate ribose (cADPR) and dopamine, visually showed spatial recovery patterns of metabolic signals following apr-YTF intervention. These findings suggest that MALDI-MSI can complement conventional biochemical and histopathological assessments by linking metabolic alterations with their spatial localization in cardiac tissue, while providing spatial clues for identifying candidate metabolic features associated with apr-YTF treatment rather than direct evidence of validated drug targets.

### 4.5 Limitations and future perspectives

This study has several limitations. First, a sham-operated control group and a positive drug control group were not included, which limits the ability to fully distinguish the effects of surgical stress and to compare apr-YTF with standard cardioprotective interventions. Second, MALDI-MSI was performed only in positive-ion mode using DHB as the matrix; therefore, some acidic lipids and nucleotide metabolites may not have been comprehensively detected. Third, strict ROI-based quantification of ischemic, border, and remote myocardial regions based on pixel-level co-registration with TTC/HE/Masson images was not performed. Therefore, the MALDI-MSI results should be interpreted as whole-section spatial distribution patterns and group-level metabolic remodeling features rather than precisely co-registered region-specific measurements. Fourth, although several metabolites were annotated by MS/MS database matching, authentic standards and targeted LC-MS/MS validation were not performed for all reversed metabolic features. Fifth, although all apr-YTF preparations used in the present study were prepared from the same batch to minimize batch-related variability, detailed batch-to-batch chemical fingerprinting was not performed. Moreover, because systematic annotation of apr-YTF-derived drug-related features was not conducted, potential overlap or interference between xenobiotic peaks derived from apr-YTF and endogenous metabolite signals in MALDI-MSI cannot be completely excluded. Sixth, the proposed cADPR/Ca^2+^-related mechanism was inferred from spatial metabolomics and remains exploratory. Complementary functional assays for calcium handling were not performed in the present study. Specifically, we did not assess ryanodine receptor 2 (RyR2) expression or activity, cluster of differentiation 38 (CD38) activity, Ca^2+^ transients, mitochondrial Ca^2+^ accumulation, or mitochondrial permeability transition pore opening. Therefore, a causal relationship among apr-YTF intervention, cADPR changes, calcium-homeostasis regulation, and cardioprotection cannot be established based on the current data. Future studies should combine chemical quality control, targeted metabolomics, drug-derived feature annotation, and functional calcium-handling experiments to further clarify the cardioprotective mechanism of apr-YTF.

## 5 Conclusion

The active parts of Yangxin Tongmai Formula ameliorated myocardial injury and were associated with partial correction of spatial metabolic remodeling in rats with MIRI.

## Data Availability

The original contributions presented in the study are included in the article. Further inquiries can be directed to the corresponding author.
